# Detrimental effects of soluble α-synuclein oligomers at excitatory glutamatergic synapses

**DOI:** 10.3389/fnagi.2023.1152065

**Published:** 2023-03-16

**Authors:** Elena Ferrari, Michela Salvadè, Elisa Zianni, Marta Brumana, Monica DiLuca, Fabrizio Gardoni

**Affiliations:** Department of Pharmacological and Biomolecular Sciences (DiSFeB) “Rodolfo Paoletti”, University of Milan, Milan, Italy

**Keywords:** Parkinson’s disease, NMDA–receptor, dendritic spine, mice, glutamate

## Abstract

**Introduction:**

Oligomeric and fibrillar species of the synaptic protein α-synuclein are established key players in the pathophysiology of Parkinson’s disease and other synucleinopathies. Increasing evidence in the literature points to prefibrillar oligomers as the main cytotoxic species driving dysfunction in diverse neurotransmitter systems even at early disease stages. Of note, soluble oligomers have recently been shown to alter synaptic plasticity mechanisms at the glutamatergic cortico-striatal synapse. However, the molecular and morphological detrimental events triggered by soluble α-synuclein aggregates that ultimately lead to excitatory synaptic failure remain mostly elusive.

**Methods:**

In the present study, we aimed to clarify the effects of soluble α-synuclein oligomers (sOligo) in the pathophysiology of synucleinopathies at cortico-striatal and hippocampal excitatory synapses. To investigate early defects of the striatal synapse *in vivo*, sOligo were inoculated in the dorsolateral striatum of 2-month-old wild-type C57BL/6J mice, and molecular and morphological analyses were conducted 42 and 84 days post-injection. In parallel, primary cultures of rat hippocampal neurons were exposed to sOligo, and molecular and morphological analyses were performed after 7 days of treatment.

**Results:**

*In vivo* sOligo injection impaired the post-synaptic retention of striatal ionotropic glutamate receptors and decreased the levels of phosphorylated ERK at 84 days post-injection. These events were not correlated with morphological alterations at dendritic spines. Conversely, chronic *in vitro* administration of sOligo caused a significant decrease in ERK phosphorylation but did not significantly alter post-synaptic levels of ionotropic glutamate receptors or spine density in primary hippocampal neurons.

**Conclusion:**

Overall, our data indicate that sOligo are involved in pathogenic molecular changes at the striatal glutamatergic synapse, confirming the detrimental effect of these species in an *in vivo* synucleinopathy model. Moreover, sOligo affects the ERK signaling pathway similarly in hippocampal and striatal neurons, possibly representing an early mechanism that anticipates synaptic loss.

## 1. Introduction

The molecular mechanisms addressing the role of α-synuclein (αSyn) aggregates in the pathophysiology of Parkinson’s disease are mostly elusive; however, a wide range of structurally diverse oligomeric and fibrillar species have been evaluated in *in vivo* and *in vitro* preclinical studies (Bousset et al., 2013; Chen et al., 2015; Pieri et al., 2016).

Prior to being deposited in insoluble inclusions, pre-amyloid αSyn aggregates can spread inter-neuronally, contributing to pathology propagation and synaptic alterations (Hansen et al., 2011; Bengoa-Vergniory et al., 2017; Cascella et al., 2022). However, studying the differential contribution of αSyn toxic aggregates in synucleinopathy progression is complicated by the broad heterogeneity of these pathological forms of the protein. Monitoring their spread and cytotoxic activity *in vivo* is challenging due to their unstable and dynamic nature (Estaun-Panzano et al., 2023). This is especially true for small soluble oligomeric species that can represent both early phases of the αSyn aggregation process or degradation by-products of larger aggregates (Kulenkampff et al., 2021; Bigi et al., 2022).

In recent years, increasing evidence has described the detrimental effects of αSyn protofibrils on various neurotransmitter systems and brain regions (Peelaerts et al., 2015; Lau et al., 2020) and the putative role of oligomeric forms as significant players in disease spread and neurodegeneration (Kulenkampff et al., 2021; Emin et al., 2022). Specifically, small soluble αSyn oligomers (sOligo), generated by *in vitro* reactions or isolated from post-mortem patients’ brains, were shown to trigger inflammation and neurotoxicity *in vitro* more efficiently than larger aggregates (Emin et al., 2022). Moreover, fibrillar forms were suggested to induce neurotoxicity, even in an indirect manner, through the release of soluble oligomers. Once taken up by neurons, these aggregates could further sustain the spread of disease through cell-to-cell transfer, seeding, and the formation of new fibrils (Cascella et al., 2022). More specifically, a large number of studies in the past decade have investigated the synaptotoxicity of synuclein aggregates (Bate et al., 2010; Rockenstein et al., 2014; Paumier et al., 2015), identifying detrimental effects not only at the presynaptic level but also at the post-synaptic compartment level (Diogenes et al., 2012; Chen et al., 2015; Ferreira et al., 2017; Sarafian et al., 2017; Trudler et al., 2021). Interestingly, recent studies demonstrated the detrimental effect of αSyn sOligo and αSyn preformed fibrils (αSyn-PFF) on glutamatergic neurotransmission at cortico-striatal synapses in early stages of disease progression, long before the onset of significant dopaminergic nigrostriatal degeneration (Tozzi et al., 2016, 2021; Durante et al., 2019). αSyn-PFF can interfere with bidirectional synaptic plasticity, preventing the induction of both long-term potentiation and long-term depression (Tozzi et al., 2021). In addition, our group recently demonstrated that αSyn-PFF induces dendritic spines loss at cortico-striatal synapses, and this event was correlated with reduced post-synaptic availability of both AMPA receptor (AMPAR) and NMDA receptor (NMDAR) subunits (Ferrari et al., 2022). Conversely, despite evidence that αSyn sOligo can cause synaptic plasticity defects of the cortico-striatal synapse (Durante et al., 2019), their role in driving synucleinopathies at the excitatory glutamatergic synapse and the precise molecular events underlying these defects remain unclear.

We investigated the role of αSyn sOligo in the events underlying early αSyn-driven synaptic dysfunction. To this, sOligo were inoculated in the dorsolateral striatum of 2-month-old wild-type C57BL/6J mice, and molecular and morphological analyses were conducted 42 and 84 days post-injection. Since its first generation in Luk et al. (2012), these rodent models have been extensively characterized to exhibit slow and progressive deterioration of the nigrostriatal tract and dissemination of αSyn neuropathology, making them suitable to reproduce the early disease phases (Henderson et al., 2019; Gómez-Benito et al., 2020). Moreover, *in vivo* experiments were replicated *in vitro* in primary hippocampal neurons to produce a more complete picture of the effect of sOligo in mediating synaptic toxicity at glutamatergic synapses in different brain areas.

## 2. Materials and methods

### 2.1. Preparation of αsyn soluble oligomers

α-synuclein oligomers were generated through a recently reported oligomerization protocol with a few modifications (Durante et al., 2019). Recombinant human αSyn monomeric protein (Proteos, Kalamazoo, MI, USA) was thawed on ice, centrifuged at 13,000 × *g* for 10 min at 4°C, and the supernatant was retained (Polinski et al., 2018). After measurement of protein concentration, the solution was diluted to a final concentration of 2 mg/ml in phosphate-buffered saline (PBS) (Sigma-Aldrich) and incubated for 2 h at room temperature (RT) under constant agitation (1,000 rpm) in a benchtop Thermomixer. The solution was then aliquoted and stored at −80°C.

### 2.2. Transmission electron microscopy validation of sOligo preparation

For each experiment, the presence of oligomeric species in the solution was evaluated by negative staining protocol with a Talos L120C transmission electron microscope (TEM) (Thermo Fisher, Waltham, MA, USA) operating at 120 kV. Digital images were acquired using a 50 CETA-MTM 4k × 4k camera (Thermo Fisher). TEM analyses were performed at the Unitech NOLIMITS imaging facility of the University of Milan.

### 2.3. Animals

Experimental procedures were conducted following the National Institutes of Health Guide for the Care and Use of Laboratory Animals, the European Community Council Directives 2010/63/EU, and the Italian law 26/2014. Approval of all procedures involving animals were obtained from the local Animal Use Committee and the Italian Ministry of Health (permits 1200/2020-PR, 330/2018-PR and 5247B.N.YCK2018). Two-month-old male C57BL/6J mice were housed in cages (two to four per cage) in a climate-controlled facility (22 ± 2°C), with *ad libitum* access to food and water throughout and a 12-h light–dark cycle (19:00–07:00 schedule). Each *ex vivo* experimental approach (spine morphology and biochemistry) was conducted using distinct cohorts of mice.

#### 2.3.1. Surgical procedures

C57BL/6J male mice were anesthetized with a mixture of isoflurane/oxygen by inhalation and mounted on a stereotaxic frame (Stoelting) linked to a digital micromanipulator. Brain coordinates for the dorsolateral striatum were chosen as previously described (Luk et al., 2012; Ferrari et al., 2022): anterior–posterior, + 0.2 mm; medial–lateral, ± 2 mm; and dorsal–ventral, – 2.6 mm. For each hemisphere, 2.5 μl of αSyn sOligo (5 μg) (Luk et al., 2012; Tozzi et al., 2021; Ferrari et al., 2022) or PBS was infused through a 10 μl Hamilton syringe using a microinjection pump at a flow rate of 0.25 μl/min. The needle was left in place at the end of the injection for 4 min to allow the solution to flow out entirely.

#### 2.3.2. Isolation of triton-insoluble post-synaptic fractions from mouse striata

To isolate Triton-insoluble post-synaptic fractions highly enriched in post-synaptic density proteins (Stanic et al., 2017), striata were homogenized with a Teflon-glass potter at 4°C in ice-cold buffer (pH 7.4) containing 0.32 M sucrose, 1 mM HEPES, 1 mM MgCl_2_, 1 mM NaHCO_3_, and 0.1 mM phenylmethanesulfonylfluoride supplemented with Complete™ protease inhibitor cocktail (Roche Diagnostics) and phosSTOP™ phosphatase inhibitor (Roche Diagnostics). An aliquot of the homogenate was frozen at −20°C and kept subsequent analysis, while the rest of the sample was spun at 13,000 × *g* for 15 min at 4°C. The resulting pellet (P2-crude membrane fraction) was resuspended in Triton-KCl buffer (1% Triton™ X-100 and 150 mM KCl). After 15 min incubation on ice, the solution was centrifuged at 100,000 × *g* for 1 h at 4°C. The pellet (triton-insoluble post-synaptic fraction) was resuspended in 20 mM HEPES buffer supplemented with Complete™ protease inhibitor cocktail tablets and stored at −80°C.

#### 2.3.3. *Ex vivo* spine morphology

For *ex vivo* confocal imaging of the dendritic spines of striatal spiny projection neurons (SPNs), brain slices were labeled with DiI dye (Invitrogen), a fluorescent lipophilic carbocyanine dye that labels spine structures and fine dendritic arborization (Kim et al., 2007). The DiI labeling procedure was performed as previously described (Stanic et al., 2015). Briefly, after mouse cardiac perfusion with 1.5% paraformaldehyde (PFA) in 0.1 M phosphate buffer, DiI solid crystals were applied with a thin needle onto the region of interest on both sides of 3-mm brain slices comprising the striatum. The DiI dye was allowed to diffuse for 16 h in the dark at RT in 0.1 M phosphate buffer; slices were subsequently post-fixed with 4% PFA in 0.1 M phosphate buffer for 45 min at 4°C. Then, 100 μm striatal slices were obtained using a vibratome and mounted on Superfrost glass slides (Thermo Fisher) with Fluoroshield (Sigma) for confocal imaging. The Zeiss Confocal LSM900 system was used to acquire fluorescence images at 555 nm with a 63X objective and Z-stack of 0.45 μm. Analysis of spine morphology and density was performed using Fiji (ImageJ) software. Specifically, spine length and width were manually measured at selected regions of interest in basal dendrites, with a distance from the cell body of up to 300 μm. A total dendritic length of about 200–300 μm from an average of three basal dendrites was considered for each neuron. For each dendritic spine, the spine head and neck width and spine length were measured, and these values were used to classify dendritic spines into three categories (thin, stubby and mushroom) [see also (Harris et al., 1992; Gardoni et al., 2012)]. In particular, the length and the ratio between the width of head and the width of neck (Wh/Wn) were used as parameters for the classification as follows: protrusions having a length of more than 3 μm were considered as filopodia (and therefore excluded from the quantitative analysis), the others as spines; spines with a Wh/Wn ratio bigger than 1.7 were considered mushrooms; spines with a Wh/Wn ratio smaller than 1.7 were divided in stubby, if shorter than 1 μm, and thin if longer than 1 μm.

### 2.4. Primary hippocampal neuronal cultures

Primary hippocampal neuronal cultures were prepared from embryonic day 18–19 (E18-E19) Sprague–Dawley rat (Charles River, Milan, Italy). In details, the hippocampi were isolated from the embryos’ brains under a dissecting microscope. Under a sterile hood, the isolated hippocampi were then washed with ice cold HBSS medium (Hank’s balanced salt solution, Sigma) and then incubated at 37°C with HBSS supplemented with trypsin for 13 min to dissociate the tissue. Tissue dissociation was then blocked by washes with plating medium (DMEM) + glutamax (Invitrogen) with 10% Horse Serum (Euroclone) and 1% Pen/Strep (Invitrogen). Finally, neurons were plated on Poli-L-Lysine (Sigma)-coated culture supports/dishes in plating medium at a density of 20,000 and 37,500 cells/cm^2^ for imaging and biochemical analyses, respectively. 16 h after plating, the medium was replaced with Neurobasal medium with 2% B27 supplement (Gibco), 1% Glutamax (Invitrogen) and 1% Pen/Strep (Invitrogen).

#### 2.4.1. sOligo treatment of primary hippocampal neurons

α-synuclein sOligo aliquots were thawed at RT and diluted in sterile PBS to a final concentration of 0.1 μg/μl. As previously reported (Wu et al., 2019), a single dose of sOligo was added at *DIV9* (9 days *in vitro*) to the neuronal culture medium at a concentration of 2 μg/ml and was left for 7 days until *DIV16*. Control neurons were treated only with the vehicle solution (PBS).

#### 2.4.2. *In vitro* spine morphology

*In vitro* spine morphology was performed as previously reported (Gardoni et al., 2012; Stanic et al., 2015; Dinamarca et al., 2016). Briefly, for *in vitro* morphological studies, neurons were transfected with an enhanced green fluorescent protein (EGFP) plasmid at *DIV7* using the calcium-phosphate coprecipitation method. Hippocampal neurons were fixed at *DIV16* for 15 min at RT in 4% PFA in PBS supplemented with 4% sucrose in PBS. Images were acquired using an inverted LSM900 confocal microscope (Zeiss) with a 63X objective (Supplementary Figure 1). Neurons were randomly acquired from different coverslips from three independent experiments and analyzed using Fiji (ImageJ) software. Specifically, spine length and width were manually measured at selected regions of interest in basal dendrites, with a distance from the cell body of up to 300 μm. A total dendritic length of about 200–300 μm from an average of three basal dendrites was considered for each neuron. For quantitative analysis, the same method described above in (Section “2.3.3. *Ex vivo* spine morphology”) was used.

#### 2.4.3. Isolation of triton-insoluble post-synaptic fractions from primary hippocampal neurons

Primary hippocampal neurons were harvested at *DIV16* by mechanical scraping in lysis buffer containing 0.32 M sucrose, 1 mM HEPES, 1 mM MgCl_2_, 1 mM NaHCO_3_, and 0.1 mM phenylmethanesulfonylfluoride supplemented with Complete™ Protease Inhibitor Cocktail Tablets (Roche Diagnostics) and phosSTOP™ Phosphatase Inhibitor (Roche Diagnostics). Cell lysates were then homogenized using a glass-glass potter in ice-cold lysis buffer, and an aliquot of the total homogenate was stored at −20°C. The sample was centrifuged at 13,000 × *g* for 15 min at 4°C, and the pellet (P2-crude membrane fraction) was resuspended in Triton-KCl buffer (0.5% Triton™ X-100 and 150 mM KCl). After 15-min incubation at 4°C, samples were spun at 100,000 × *g* for 1 h at 4°C. The pellet (TIF) was resuspended in 20 mM HEPES buffer supplemented with Complete™ protease inhibitor cocktail tablets and stored at −80°C.

### 2.5. SDS-PAGE and Western blotting

The Bradford assay was used to determine the protein content of the homogenate and TIF samples from striatal and primary hippocampal neurons. Samples were then denatured chemically with Laemmli buffer and thermally by heating at 98°C for 10 min. For Western blotting analysis, a total of 10–25 μg of protein was separated by sodium dodecyl sulfate-polyacrylamide gel electrophoresis on 6–12% acrylamide/bisacrylamide gel and transferred to a nitrocellulose membrane (Biorad) by electroblotting. Membranes were blocked for 1 h at RT in blocking solution (I-block, TBS 1X, and 20% Tween-20) on a shaker and incubated with the specific primary antibody diluted in blocking solution overnight at 4°C. The next day, the membranes were washed three times in TBSt (TBS and 0.1% Tween20) and subsequently incubated with the corresponding horseradish peroxidase-conjugated secondary antibody diluted in blocking solution for 1 h at RT. After three washes with TBSt, the membranes were developed with electrochemiluminescence reagents (Biorad) and scanned with a Chemidoc (Biorad Universal Hood III) using Image Lab software (Biorad). Bands were quantified with computer-assisted imaging (Image Lab, Biorad). Protein levels were expressed as relative optical density measurements normalized to a housekeeping protein.

### 2.6. Antibodies

The primary antibodies used were: mouse anti-tubulin (1:30,000, #T9026, Sigma), rabbit anti-GAPDH (1:5,000, #sc-25778, Santa Cruz), rabbit anti-GluN2A (1:1,000, #M264, Sigma), rabbit anti-GluN2B (1:1,000, #718600, Invitrogen), mouse anti-GluN2D (1:1,000, #MAB5578, Millipore), rabbit anti-GluA1 (1:1,000, #13185, Cell Signaling), mouse anti-GluA2 (1:1,000, #75–002, Neuromab), mouse anti-GluA3 (1:1,000, #MAB5416, Millipore), polyclonal anti-Rph3A (1:2,000, Protein Tech, #11396-1-AP), mouse anti-PSD-95 (1:1,000, #K28/43, Neuromab), rabbit anti-tyrosine hydroxylase (1:10,000, #AB152, Millipore), rabbit anti-phospho-extracellular signal-regulated kinase (ERK) 44/42 (1:1,000, Cell Signaling, #9101), rabbit anti-ERK 44/42 (1:1,000, #9102, Cell Signaling), rabbit anti-phospho-cAMP responsive element binding protein (CREB) (1:1,000, #9198, Cell Signaling), and rabbit anti-CREB (1:1,000, #9197, Cell Signaling). The secondary horseradish peroxidase-linked antibodies used were goat anti-rabbit (1:10,000, #172-1019, Biorad) and goat anti-mouse (1:10,000, #172-1011, Biorad).

### 2.7. Statistical analyses

Image Lab software (Biorad) was used for the quantification of Western blot experiments. Protein levels are reported as relative optical density normalized on tubulin/GAPDH as housekeeping proteins. Images acquired through confocal microscopy were analyzed using Fiji (ImageJ) software.

Statistical analyses were performed using GraphPad Prism 8 software, and data are presented as mean ± standard error of the mean (SEM). The figure legends include the tests used to evaluate the statistical data significance. Specifically, the following tests were used, as appropriate: two-tailed unpaired Student’s *t*-test, Mann–Whitney test, one-way ANOVA, and Kruskal–Wallis test. The numbers of neurons and mice used in each experiment are described in the figure legends.

## 3. Results

### 3.1. Post-synaptic molecular alterations of the cortico-striatal synapse induced by *in vivo* striatal injection of sOligo

**FIGURE 1:**
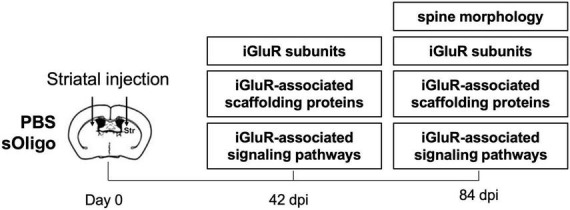
Schematic representation of the experimental procedures.

**FIGURE 2:**
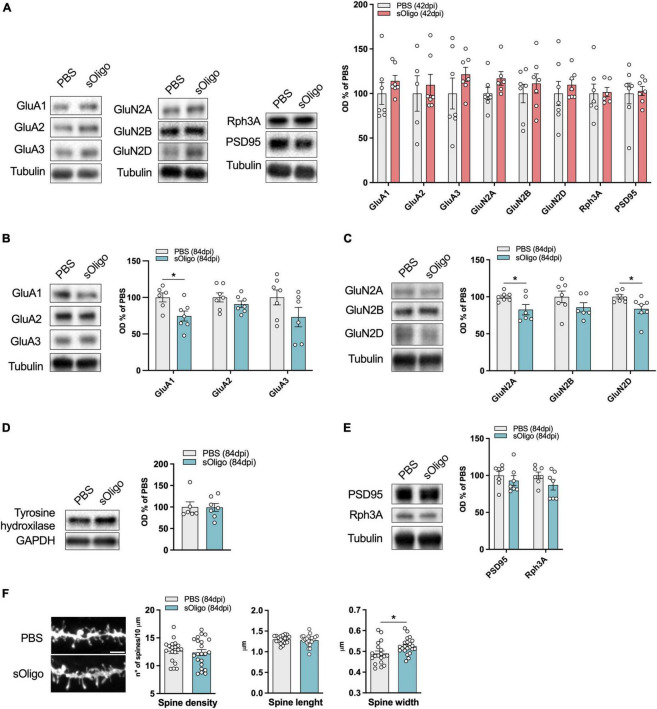
Molecular effects induced by *in vivo* striatal injection of αSyn soluble oligomers in mice at the corticostriatal synapse. **(A)** Post-synaptic levels of AMPAR (GluA1, GluA2, and GluA3) and NMDAR (GluN2A, GluN2B, and GluN2D) subunits and scaffolding proteins (Rph3A and PSD95) were evaluated by Western blot in striatal TIF of sOligo- and PBS-injected mice 42 dpi. Protein levels normalized on tubulin were reported as OD% of PBS-mice. *n* = 5–7 mice. Post-synaptic levels of **(B)** AMPAR (GluA1, GluA2, and GluA3) subunits, **(C)** NMDAR (GluN2A, GluN2B, and GluN2D) subunits and **(E)** scaffolding proteins (Rph3A and PSD95) were evaluated by Western blot in striatal TIF of sOligo- and PBS-injected mice 84 dpi. Protein levels normalized on tubulin were reported as OD% of PBS-mice. *n* = 5–7 mice. **(D)** Expression of the dopaminergic marker Tyrosine hydroxylase was evaluated by Western blot in striatal homogenates of sOligo- and PBS-injected mice 84 dpi. Protein levels normalized on GAPDH were reported as OD% of PBS-mice. *n* = 7 mice. **(F)** Representative confocal images and quantification of spine morphology analyses (spine density, spine length and spine width) of SPNs of sOligo- and PBS-injected mice 84 dpi. Scale bar: 3 μm. *n* = 19–22 neurons from 3 mice. Data are represented as mean ± SEM. *P < 0.05 (Student’s *t*-test; data with non-normal distribution were tested with Mann–Whitney test).

α-synuclein oligomers were prepared using a validated protocol with some modifications [see section “2. Materials and methods”; (Polinski et al., 2018; Durante et al., 2019)]. The presence and the morphology of sOligo was assessed by TEM prior to their experimental use (Supplementary Figure 2). To characterize the impact of sOligo species at the cortico-striatal synapse, we injected sOligo into the dorsolateral striatum of 2-month-old mice (Figure 1). Control mice were injected with PBS, the vehicle of αSyn oligomerization. Taking into account previous studies addressing the detrimental effects of αSyn aggregates on glutamatergic synapses (Tozzi et al., 2021; Ferrari et al., 2022), molecular analyses were conducted at 42 and 84 days post-injection (dpi). We first investigated the molecular composition of the striatal glutamatergic post-synaptic compartment in terms of ionotropic glutamatergic receptor (iGluR) levels. To this aim, TIFs enriched in PSD proteins were extracted from mouse striata and iGluR expression was assessed by Western blotting. At 42 dpi, post-synaptic levels of AMPAR subunits GluA1, GluA2, and GluA3 and NMDAR subunits GluN2A, GluN2B, and GluN2D were unchanged in sOligo mice compared with the controls (Figure 2A). Interestingly, these findings indicate a lack of toxicity of sOligo on post-synaptic iGluRs at 42 dpi, mirroring what previously reported following *in vivo* PFF-seeding (Ferrari et al., 2022). Considering the fundamental role of scaffolding proteins in regulating post-synaptic retention of iGluRs, we subsequently measured the post-synaptic levels of the most abundant PSD-associated protein, PSD-95, and the NMDAR-scaffolding partner, Rph3A, which were recently demonstrated to contribute to early synaptic dysfunction in experimental models of synucleinopathies induced by αSyn-PFF (Stanic et al., 2015; Ferrari et al., 2022). However, no alterations were observed for either scaffolding element at this time point (Figure 2A).

In contrast to the observations at 42 dpi, sOligo-injected mice exhibited a decrease in both AMPAR and NMDAR synaptic levels at 84 dpi (Figures 2B, C). In particular, we observed a significant decrease in the AMPAR-GluA1 subunit accompanied by a trend in decrease of the GluA2 and GluA3 subunits compared with PBS mice (Figure 2B). In addition, as shown in Figure 2C, levels of the NMDAR subunits GluN2A and GluN2D were significantly decreased upon oligomeric αSyn seeding. Again, these alterations in iGluR expression at 84 dpi mirrored findings in the PFF-mouse model (Ferrari et al., 2022), suggesting generalized toxicity of diverse αSyn species toward glutamatergic transmission that anticipates neurodegeneration. However, sOligo inoculation did not affect the integrity of dopaminergic striatal afferents, as shown by unaltered striatal tyrosine hydroxylase expression in sOligo-treated mice compared with controls (Figure 2D). Moreover, post-synaptic expression of Rph3A showed a trend in decrease in sOligo-mice compared with controls, while no significant change in the level of PSD-95 was detected (Figure 2E). Finally, no modifications of AMPAR and NMDAR subunits were observed in striatal homogenates at dpi 84 thus suggesting an impairment of their synaptic localization in absence of alterations of their total protein levels (Supplementary Figure 3).

We next investigated whether the molecular impairments of iGluRs were reflected in alterations of dendritic spine morphology of SPNs, the most abundant neuronal type of the striatum (Graveland and Difiglia, 1985). At 84 dpi, sOligo-mice displayed no variation in dendritic spine density compared to PBS mice, as reported by *ex vivo* morphological analyses (Figure 2F). SPNs of treated mice displayed normal spine length but a significant increase in spine width (Figure 2F), which could underlie compensatory mechanisms to the previously described modifications at the receptor level.

**FIGURE 3:**
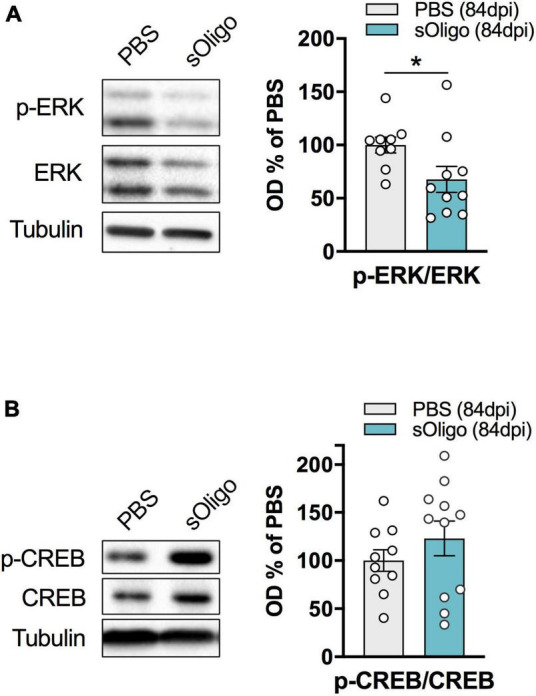
Effects of *in vivo* striatal injection of αSyn soluble oligomers in mice on ERK and CREB signaling. **(A)** Levels of pERK were evaluated by Western blot in striatal homogenates of sOligo- and PBS-injected mice 84 dpi. pERK level was normalized on total ERK expression and reported as OD% of PBS-mice. *n* = 9–10 mice. **(B)** Levels of pCREB were evaluated by Western blot in striatal homogenates of sOligo- and PBS-injected mice 84 dpi. pCREB level was normalized on total CREB expression and reported as OD% of PBS-mice. *n* = 10–11 mice. Data are represented as mean ± SEM. *P < 0.05 (Student’s *t*-test; data with non-normal distribution were tested with Mann–Whitney test).

Considering the observed alteration of post-synaptic levels of iGluRs at 84 dpi, we evaluated possible modifications of downstream signaling pathways at this time point. Synaptic NMDAR activity promoted nuclear signaling to CREB, regulating gene expression of pro-survival factors and anti-apoptotic pathways together with the activity of the ERK (Hardingham and Bading, 2010). Therefore, the levels of phosphorylated ERK (p-ERK) and CREB (p-CREB) were assessed by Western blot analysis in the striatal homogenate. Interestingly, upon sOligo seeding, levels of p-ERK were significantly reduced compared with controls (Figure 3A). In contrast, no significant change in p-CREB level was detected at the same time point (Figure 3B). Finally, in accordance with the results about NMDAR and AMPAR synaptic localization (Figure 2A), no alterations of p-ERK levels were detected at dpi 42 (Supplementary Figure 4).

To summarize, at 84 dpi, *in vivo* sOligo seeding caused a reduction in the amount of iGluR complexes at the spines, primarily affecting the AMPAR GluA1 and the NMDAR GluN2A and GluN2D subunits, without altering the number of synaptic contacts. A decrease in p-ERK in these mice suggests that αSyn possibly affects downstream signaling pathways related to ERK activity. Moreover, the concomitant increase in dendritic spine width could represent an early compensatory mechanism to overcome the cortico-striatal signaling defects induced by sOligo.

### 3.2. Morphological and molecular effects induced by αSyn sOligo at the hippocampal synapse *in vitro*

**FIGURE 4:**
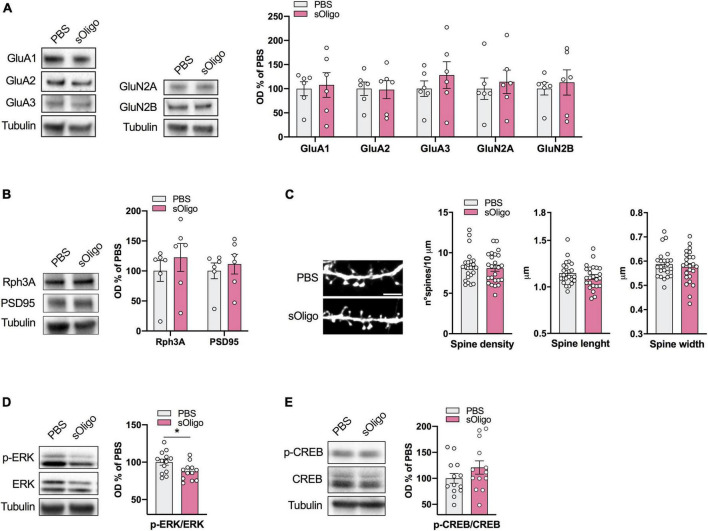
Molecular effects induced by αsyn sOligo at the hippocampal synapse *in vitro*. Primary cultures of rat hippocampal neurons were treated at DIV9 with either 2 μg/μl sOligo or PBS and analyses were performed at DIV16. Post-synaptic levels of **(A)** AMPAR (GluA1, GluA2, and GluA3) and NMDAR (GluN2A and GluN2B) subunits and **(B)** the scaffolding protein Rph3A and PSD-95 were evaluated by Western blot in TIF from sOligo- or PBS-neurons. Protein levels normalized on tubulin were reported as OD% of PBS-neurons. *n* = 6 independent cultures. **(C)** Representative confocal images and quantification of spine morphology analyses (spine density, spine length and spine width) of sOligo- and PBS-neurons at DIV16. Scale bar: 3 μm. *n* = 23–24 neurons from 3 independent cultures. **(D)** Levels of pERK were evaluated by Western blot in homogenates of sOligo- and PBS-neurons at DIV16. pERK level was normalized on total ERK expression and reported as OD% of PBS-neurons. *n* = 12–13 independent cultures. **(E)** Levels of pCREB were evaluated by Western blot in homogenates of sOligo- and PBS-neurons at DIV16. pCREB level was normalized on total CREB expression and reported as OD% of PBS-neurons. *n* = 14 independent cultures. Data are represented as mean ± SEM. *P < 0.05 (Student’s *t*-test; data with non-normal distribution were tested with Mann–Whitney test).

Data collected using the *in vivo* sOligo-mouse model suggest that soluble αSyn species affect striatal post-synaptic molecular architecture and signaling. To further evaluate possible widespread sOligo toxicity toward glutamatergic neurotransmission, we aimed to evaluate the effect of sOligo on the hippocampal excitatory synapse. To this end, we used primary hippocampal neuronal cultures, a highly validated model for the assessment of synaptic structure and function (Dailey and Smith, 1996; Wu et al., 2019). Hippocampal cultures have been widely exploited to study the modulation of neuronal and synaptic homeostasis following acute and chronic αSyn exposure (Chen et al., 2015; Ferreira et al., 2017; Wu et al., 2019; Shrivastava et al., 2020). In addition, in line with previous findings, we recently demonstrated that 7-day exposure to αSyn-PFFs (2 μg/ml) induced diminished post-synaptic expression of GluN2A-containing NMDARs, leading to early spine loss (Ferrari et al., 2022). Therefore, taking the *in vitro* PFF-neuronal model as a reference, hippocampal primary neurons were treated at *DIV9* by administration of the αSyn sOligo preparation (2 μg/ml) in the culture medium. Control neurons were treated with the vehicle of αSyn solution (PBS). We first evaluated the composition of the glutamatergic post-synaptic compartment of treated hippocampal neurons at *DIV16*. As shown in Figure 4A, 7-day administration of sOligo did not significantly alter post-synaptic levels of the AMPAR GluA1, GluA2, and GluA3 subunits and the NMDAR GluN2A and GluN2B subunits, which are the most abundant iGluR subunits in the hippocampus. In line with these data, no alterations in the levels of scaffolding proteins Rph3A and PSD-95 were found in sOligo-treated neurons compared with controls (Figure 4B). To assess the possible morphological effects of sOligo at dendritic spines at the same time point, hippocampal neurons were transfected with enhanced green fluorescent protein at *DIV7* and subsequently treated with sOligo preparation or PBS at *DIV9*. *In vitro* spine morphology analysis indicated no significant alterations in spine density at *DIV16* upon sOligo exposure compared with PBS administration (Figure 4C). Moreover, sOligo-treated neurons displayed normal spine length and width when compared to controls (Figure 4C), suggesting a lack of early synaptic toxicity of soluble species in this experimental setting. These findings indicate that, at the time point and dose evaluated, soluble αSyn species are incapable of triggering post-synaptic molecular or morphological alterations at hippocampal excitatory synapses, which contrasts what was recently reported in the αSyn-PFF *in vitro* model (Wu et al., 2019; Ferrari et al., 2022).

Because we detected the impact of sOligo on striatal ERK signaling *in vivo* (Figure 3A), we also evaluated p-ERK and p-CREB protein levels in the hippocampal neuronal homogenate by Western blot analysis. In agreement with the *in vivo* data, *in vitro* sOligo administration to neurons induced a decrease in p-ERK at *DIV16* (Figure 4D). In contrast, p-CREB levels were not significantly affected by αSyn treatment (Figure 4E).

## 4. Discussion

Few studies are currently available regarding the detrimental effect of αSyn sOligo on post-synaptic glutamate receptors (Hüls et al., 2011; Diogenes et al., 2012; Ferreira et al., 2017; Yu et al., 2019). These reports are mainly focused on the *in vitro* effect of sOligo on rat (Diogenes et al., 2012; Ferreira et al., 2017) or mice (Hüls et al., 2011; Chen et al., 2015) hippocampal synapses and demonstrate that sOligo can alter excitatory synaptic plasticity acting on AMPARs or NMDAR function at glutamatergic synapses. More recently, small αSyn aggregates were reported to reduce the post-synaptic NMDAR-mediated current and impair long-term cortico-striatal potentiation of SPNs (Durante et al., 2019). In the present work, we aimed to elucidate the impact of these species on the molecular and structural organization of dendritic spines both *in vivo* at the cortico-striatal synapse and *in vitro* at the hippocampal synapse. Specifically, pre-fibrillar aggregates (sOligo) were injected into the dorsolateral striata of mice and administered to primary hippocampal neurons.

Here we show that sOligo-injected mice exhibited significant impairments in the molecular composition of the striatal synapse at 84 dpi. In agreement with previous studies (Durante et al., 2019), we observed that αSyn sOligo impacted the striatal post-synaptic availability of GluN2A-containing NMDARs, which are known to be enriched at striatal SPN synapses. However, the significant decrease in the levels of the GluN2D subunit, known to be selectively expressed at cholinergic interneurons (Bloomfield et al., 2007; Mellone et al., 2019), highlighted an overall impairment of NMDARs that was not restricted to striatal SPNs. The toxicity of αSyn toward GluN2D-containing NMDARs was previously suggested in the adeno-associated virus-based αSyn overexpression model and was recently confirmed in mice injected with αSyn-PFF (Tozzi et al., 2016; Ferrari et al., 2022).

In addition to defects in the composition of NMDARs, sOligo mice exhibited significantly decreased expression of the GluA1 subunit of AMPARs. Impaired AMPARs signaling due to decreased synaptic GluA1 localization induced by αSyn mutants has previously been described *in vivo* (Teravskis et al., 2018). Notably, the *in vivo* impairment of the post-synaptic AMPAR and NMDAR levels resembled the alterations caused by striatal injection of αSyn-PFF (Ferrari et al., 2022). In addition, the lack of a post-synaptic effect in both sOligo and PFF mice at dpi 42 suggests that the two diverse aggregated species share a similar timing of toxicity *in vivo* (Luk et al., 2012; Ferrari et al., 2022). However, while PFF mice exhibited a concurrent significant loss of dendritic spines at striatal SPNs at dpi 84 (Ferrari et al., 2022), sOligo inoculation did not lead to synapse loss, and dendritic spines showed only a significantly increased spine head width (Ferrari et al., 2022), possibly underlying a compensatory strategy to overcome early receptor defects. Overall, the data collected on the two αSyn animal models indicate the possible differential toxicity of sOligo and PFF, namely, more severe synaptic dysfunction leading to spine loss induced by PFF at the same time point. This phenomenon could be ascribed to a greater capability of PFFs for transmitting pathology, possibly due to their release of smaller degradation products, such as oligomers. Interestingly, a similar effect was reported comparing the *in vivo* impact of β-sheet oligomers and short fibrils on the dopaminergic system. The authors observed more robust neurotoxicity and a more severe disease phenotype induced by fibrillar fragments than oligomers, supporting the greater efficiency of fibrils in spreading the pathology (Froula et al., 2019).

A large number of possible molecular mechanisms could be involved in the sOligo-mediated effect we observed on synaptic AMPA and NMDA receptors. Several studies identified αSyn docking sites at the excitatory synapses (Shrivastava et al., 2015; Ferreira et al., 2017; Matiiv et al., 2022) and described putative mechanisms mediating αSyn internalization into neurons (Shrivastava et al., 2015; Ihse et al., 2017; Delenclos et al., 2019). Interestingly, other reports put forward the hypothesis of a specific activity of αSyn in promoting NMDAR internalization through different type of molecular events (Cheng et al., 2011; Chen et al., 2015; Navarria et al., 2015; Yang et al., 2020). In this regard, our group very recently showed that αSyn can directly interact with the NMDAR scaffolding protein Rph3A leading to a reduced membrane retention of GluN2A-containing NMDARs (Ferrari et al., 2022). In line with all these previous observations, here we show that *in vivo* intra-striatal injection of sOligo reduces at dpi 84 both AMPAR and NMDAR subunits at post-synaptic sites. Interestingly, we did not observe alterations of receptors’ localization at synapses in primary hippocampal neurons treated with sOligo, thus putting forward the hypothesis of a different sensitivity of specific neuronal populations to αSyn.

Phosphorylation and activity of the mitogen-activated protein kinase ERK and the transcription factor CREB play a fundamental role in coupling synaptic NMDAR and AMPAR function to transcriptional changes underlying many neuroplasticity and pro-survival mechanisms (Wang et al., 2007). Aberrant activation of the ERK pathway was detected in late stages of Parkinson’s disease and was correlated with the onset of dyskinesia (Santini et al., 2012). We found decreased levels of p-ERK in mice injected with sOligo at 84 dpi, indicating synaptic signaling defects compatible with decreased post-synaptic expression of NMDAR and AMPAR subunits. Interestingly, 7-day treatment of primary hippocampal neurons with sOligo induced a similar decreased phosphorylation of ERK in the absence of significant alterations in spine density and synaptic localization of NMDAR and AMPAR subunits. Of note, the loss of dendritic spines in cerebrocortical slices was observed upon application of a significantly higher αSyn sOligo concentration responsible for extra-synaptic NMDAR activation (Trudler et al., 2021). Interestingly, we recently demonstrated that 7-day exposure to αSyn-PFFs triggered spine loss coupled with decreased synaptic availability of GluN2A-containing NMDARs (Ferrari et al., 2022). Overall, these data further support different and milder toxicity mechanisms induced by sOligo compared with αSyn-PFF both *in vitro* and *in vivo*.

In conclusion, our data indicate that sOligo are involved in pathogenic molecular changes at the excitatory glutamatergic synapse, confirming the detrimental effect of these αSyn species in an *in vivo* synucleinopathy model. Moreover, sOligo affects the ERK signaling pathway similarly in hippocampal and striatal neurons, possibly representing an early mechanism that anticipates synaptic loss.

## Data availability statement

The raw data supporting the conclusions of this article will be made available by the authors, without undue reservation.

## Ethics statement

The animal study was reviewed and approved by the Animal Use Committee of University of Milano and the Italian Ministry of Health.

## Author contributions

MD and FG contributed to the conception and design of the study. EF performed the statistical analysis. EF and FG wrote the first draft of the manuscript. EF, MS, EZ, and MB performed the *in vitro* and *in vivo* experiments. All authors contributed to manuscript revision, read, and approved the submitted version.
