# Supplementary material for: Detrimental effects of soluble α-synuclein oligomers at excitatory glutamatergic synapses
Source: Front Aging Neurosci. 2023 Mar 16;15:1152065. doi: 10.3389/fnagi.2023.1152065 (PMC10060538; doi:10.3389/fnagi.2023.1152065)
Supplement: Supplementary file 1 [file Image_1.PDF]

Supplementary Figure 1

EGFP-tagget primary hippocamapl neurons (DIV16)

PBS

sOligo

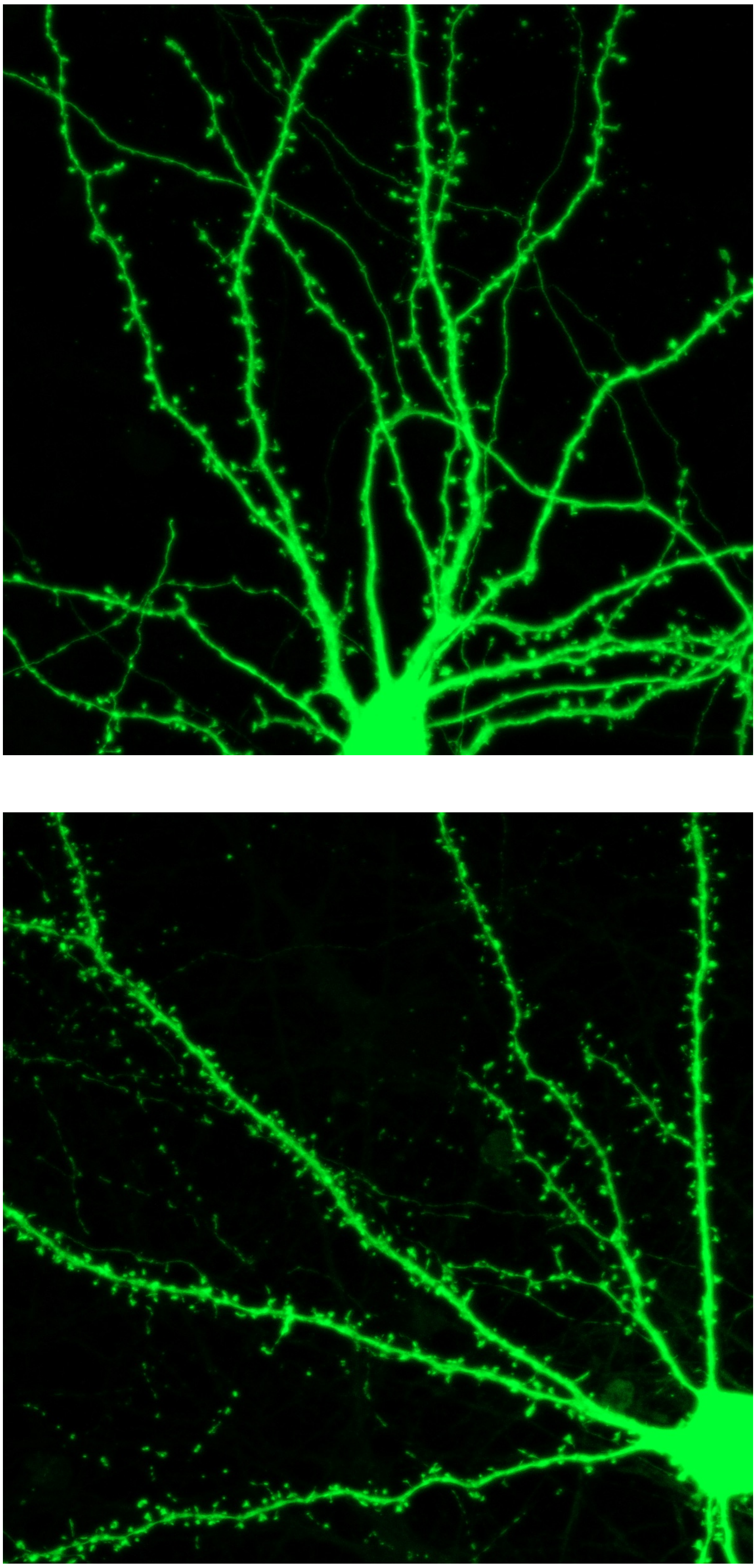

Suppl. Fig1. Representative confocal images of EGFP-tagged primary hippocampal neurons exposed to sOligo or vehicle (PBS) showing no gross modification of neuronal morphology upon sOligo treatment.

Supplementary Figure 2

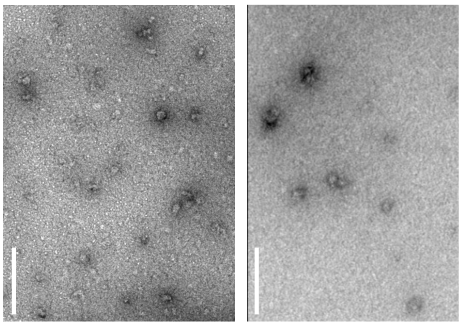

sOligo #1 sOligo #2

Suppl. Fig2. Representative images of TEM characterization of two different sOligo preparations. Scale bar: 100nm

Supplementary Figure 3

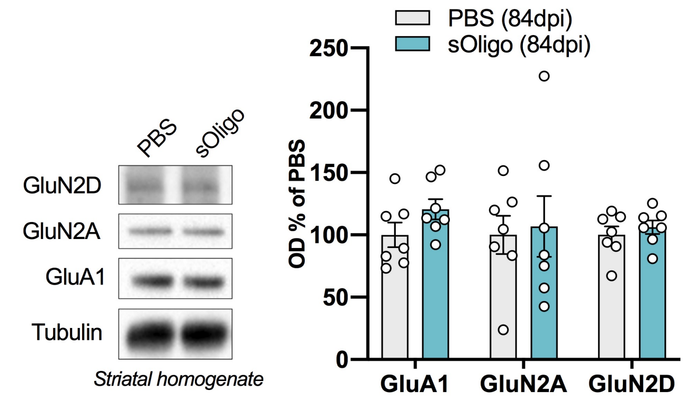

Suppl. Fig3. Effects of *in vivo* striatal injection of sOligo in mice on AMPAR-GluA1, NMDAR-GluN2A and GluN2D subunits. Total expression of GluA1, GluN2A and GluN2D subunits were evaluated by Western blot in striatal homogenates of sOligo- and PBS-injected mice 84dpi. Protein levels normalized on tubulin were reported as OD% of PBS-mice. n=7 mice. Data are represented as mean ± SEM. Mann-Whitney test, ns.

Supplementary Figure 4

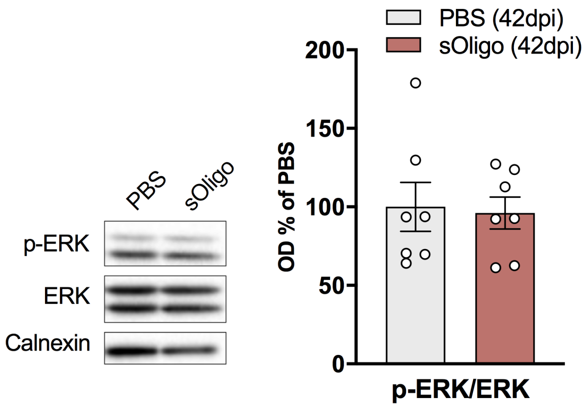

Suppl. Fig4. Effects of *in vivo* striatal injection of sOligo in mice on ERK signaling 42dpi. Levels of p-ERK were evaluated by Western blot in striatal homogenates of sOligo- and PBS-injected mice 42dpi. pERK level was normalized on total ERK expression and reported as OD% of PBS-mice. n=7 mice. Data are represented as mean ± SEM. Mann-Whitney test, ns.
